# Comparison of high- and low equipment fidelity during paediatric simulation team training: a case control study

**DOI:** 10.1186/1472-6920-14-221

**Published:** 2014-10-18

**Authors:** Lisbet Meurling, Leif Hedman, Karl-Johan Lidefelt, Cecilia Escher, Li Felländer-Tsai, Carl-Johan Wallin

**Affiliations:** Division of Anaesthesia and Intensive care, Department of Clinical Science, Intervention and Technology (CLINTEC), Karolinska Institutet, Stockholm, SE 141 86 Sweden; Division of Orthopaedics and Biotechnology, Department of Clinical Science, Intervention and Technology (CLINTEC), Karolinska Institutet, Stockholm, SE 141 86 Sweden; Division of Paediatrics, Karolinska Institutet, Department of Clinical Science, Intervention and Technology (CLINTEC), Karolinska Institutet, Stockholm, SE 141 86 Sweden; Center for Advanced Medical Simulation and Training (CAMST), Karolinska University Hospital, Stockholm, Sweden; Department of Psychology, Umeå University, Umeå, Sweden

**Keywords:** Low-fidelity, High-fidelity, Simulation, Team training, Leader, Follower, Trainer, Trainees, Mental strain, Paediatric

## Abstract

**Background:**

High-fidelity patient simulators in team training are becoming popular, though research showing benefits of the training process compared to low-fidelity models is rare. We explored in situ training for paediatric teams in an emergency department using a low-fidelity model (plastic doll) and a high-fidelity paediatric simulator, keeping other contextual factors constant. The goal was to study differences in trainees’ and trainers’ performance along with their individual experiences, during in situ training, using either a low-fidelity model or a high-fidelity paediatric simulator.

**Methods:**

During a two-year period, teams involved in paediatric emergency care were trained in groups of five to nine. Each team performed one video-recorded paediatric emergency scenario. A case control study was undertaken in which 34 teams used either a low-fidelity model (n = 17) or a high-fidelity paediatric simulator (n = 17). The teams’ clinical performances during the scenarios were measured as the time elapsed to prescribe as well as deliver oxygen. The trainers were monitored regarding frequency of their interventions. We also registered trainees’ and trainers’ mental strain and flow experience.

**Results:**

Of 225 trainees’ occasions during 34 sessions, 34 trainer questionnaires, 163 trainee questionnaires, and 28 videos, could be analyzed. Time to deliver oxygen was significantly longer (p = 0.014) when a high-fidelity simulator was used. The trainees’ mental strain and flow did not differ between the two types of training. The frequency of trainers interventions was lower (p < 0.001) when trainees used a high-fidelity simulator; trainers’ perceived mental strain was lower (<0.001) and their flow experience higher (p = 0.004) when using high-fidelity simulator.

**Conclusions:**

Levels of equipment fidelity affect measurable performance variables in simulation-based team training, but trainee s’ individual experiences are similar. We also note a reduction in the frequency of trainers’ interventions in the scenarios as well as their mental strain, when trainees used a high-fidelity simulator.

## Background

Team coordination is vital for patient safety [1, 2]. Team training can improve a team’s performance and the individual members’ teamwork behaviour and attitude towards teamwork according to research conducted in different settings [3, 4]. For non-technical skills, such as team coordination, decision making and communication, practice-based training is the most effective method [5]. Simulation is commonly used in team training in health care. Simulation fidelity has been defined in different ways. One typology is equipment-, environment-, and psychological fidelity, the last of which is generally considered the most important for training [6, 7]. Psychological fidelity refers to the degree to which trainees perceive the simulation as a reliable substitute for the actual task, or to the match between the trainee´s performance in the simulated scenario and in the real case [7]. Given a well designed team training programme, full mission simulations; i.e. high-fidelity in equipment, environmental and psychological view, are recommended for training in teamwork related skills under conditions of ambiguity, time pressure and stress [7]. According to a review by Norman et al. the evidence justifying the increased costs for equipment fidelity is limited [8]. On the other hand, Crofts et al. showed that training for shoulder dystocia using high-fidelity equipment was associated with higher successful delivery rate, shorter head – to body delivery time and a reduction in total applied force on a simulator [9]. Regarding technical skills, haptic feed-back has been shown to be an important factor for skills acquisition in the early training phase [10]. In this study low- fidelity model refers to technical equipment that is static and does not interact with the environment in contrast to high-fidelity, i.e. a mannequin that provides physiological feedback via interactive software in response to the trainees’ actions. It is a well-known among trainers, though it has not been studied as far as we know, that orally conveyed physiological parameters and practical procedures tend to pass unrealistically quickly during training with low-fidelity mannequins. The main idea behind high-fidelity is to improve the degree of realism. High fidelity patient simulators usually present physiological values on screens and provide the possibility to actually carry out practical procedures. The improvement in fidelity is likely to be reflected in a more realistic time spent for searching clinical information and carrying out procedures. The need for trainers to timely provide clinical information is also likely to be less using high fidelity equipment, and this too can affect some of the trainees’ and the trainers’ individual experiences. Team training involves not only leaders but also followers, the different roles entailing different behaviours [11–14]. Consequently, in studying the teams’ performance and individual experiences in a team, it is important to consider both leaders and followers separately [15].

Motivation is a prerequisite for learning. Research suggests that the experience of flow is a powerful motivating force and that there is a relationship between flow and skill development [16]. Flow is a subjective state when the person feels completely involved in something, forgetting time and fatigue, where the preconditions are a clear set of goals, a balance between perceived challenges and skills, and immediate feedback [16].

Another individual experience relevant for learning is mental strain (or mental effort).

A very high level of short-term mental strain may negatively influence one’s working memory [17] and hamper learning.

### Objectives

Our aim was to study trainees’ (leaders’ and followers’) and trainers’ (teachers’ or facilitators’) performance, mental strain and flow experience during in situ team training using either a low-fidelity model (LFM) or a high-fidelity paediatric simulator (HFS). Trainees are commonly studied and assessed. However, the trainers that facilitate team training scenarios are also important for learning and gaining proficiency. Our hypothesis was that training with a high-fidelity simulator would improve the experience of realism for the trainees and facilitate the trainers’ task, resulting in different behaviour and individual experiences.

## Methods

The Simulation Based Team Training was carried out in the Emergency Department at Karolinska University Hospital Huddinge, which accepts both adult and paediatric patients. The hospital has approximately 1000 beds and houses the Centre for Advanced Medical Simulation and Training (CAMST). In the Emergency Department and the Department for Paediatrics an in situ teamwork training programme for the staff using an LFM had been implemented, supervised and performed by the main trainer in this study (KJL). In 2008 a forthcoming exchange of the LFMs to HFSs provided the opportunity for a case control study.

The study was planned and performed in cooperation with the Emergency Department, the Department for Paediatrics and CAMST. Data sampling started in October 2008 and ended in May 2011.

The institutional review board (Regionala etikprövningsnämnden in Stockholm) approved the study. Trainees were offered to participate in the study and written consent was obtained (to use the recorded video-tape and questionnaires). Participants used labelled shirts in scenarios and their questionnaires were given a personal code in order to make confidential pairing of data for role in a scenario with individual experiences, age, sex, profession and earlier training possible*.* The trainees were not informed that the times for ordering and delivery of oxygen were registered, and similarly, the trainers were not informed that their interventions in the scenarios were registered.

### Ethical approval

This study was approved by The institutional review board (Regionala etikprövningsnämnden in Stockholm).

### Simulation-based team training

#### Trainees

The staff members involved in paediatric emergencies; nurses, nurse assistants, consultant paediatricians and residents were scheduled for the training sessions during their ordinary duty. The staff could be scheduled to this training more than once owing to logistic reasons. The composition of participants during each training session always varied, but some members in the team may thus have already participated in the present training or in some other kind of simulation training. None of the authors participated in the training as trainee.

#### Trainers

All trainers were physicians experienced in scenario sessions and paediatric emergency care.

#### Logistics and setting

One or two training sessions, each lasting 90 minutes, were undertaken each month during the study period of six semesters. The training was performed in situ in an ordinary emergency room equipped for paediatric patients. Video-recording equipment was temporarily installed for each session.

#### Training curriculum

The trainees trained in groups of five to nine that mixed physicians, nurses and nurse assistants corresponding to the team on duty. The trainer asked one trainee in each session to be the leader. The team assembled in the emergency room, where the trainer demonstrated the equipment for paediatric emergencies, introduced the case and encouraged team members to co-operate and communicate as they would in an authentic emergency situation. The trainees were allowed to make plans with each other and to prepare. They performed one emergency scenario, which was followed by a short debriefing and discussion.

#### Simulator

The LFMs used were ordinary dolls, one baby and one small child. During the scenario the trainer narrated the patient’s physiological state, blood pressure, pulse rate and so on.

The HFSs used were paediatric patient simulators (PediaSIM ECS and BabySIM ECS, METI Inc., Sarasota, Florida, USA). When the HFSs were used, a technician was present in one corner of the Emergency Room behind a one-way screen. The varying physiologic parameters were presented on the screen next to the patient. The trainer answered trainees’ questions about actual skin colour and moisture. There were no major difference in hardware, such as facial anatomy, sex, skin color or size of manikin, between the LFMs and HFSs.

#### Clinical cases

Two standardised paediatric emergency clinical cases were used during training: a baby/child suffering from septic shock and a baby/child suffering from severe asthma with respiratory arrest.

### Design of the study

**Figure 1:**
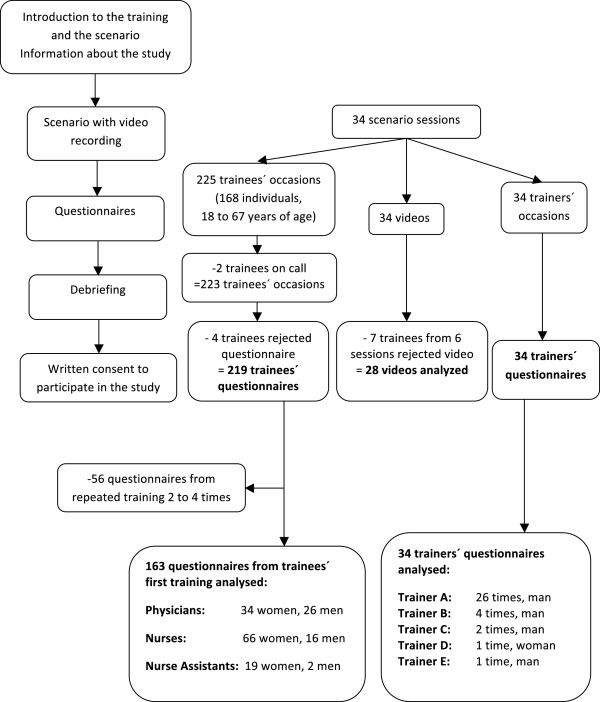
**Flow sheet for the training and videos/questionnaires received.** Thirty-four teams were trained following the same schedule. The figure presents the number of questionnaires and videos received and analysed.

The design of this case (HFS) -control (LFM) study and the subjects are presented in Figure 1. Data were collected from all training sessions during the period October 2008 - May 2011.

### Measurements

#### Clinical performance

From the videotapes, the teams’ clinical performance was measured as the time elapsed (s) from scenario’s start until oxygen was prescribed and as the time elapsed (s) from scenario’s start until oxygen was delivered. The measurements were taken independently by one researcher and thereafter a second researcher confirmed the results. The clinical performance was considered a team effort.

#### Mental strain

Immediately after each scenario, participants were asked to relate the intensity of mental strain experienced to the maximal mental strain they had experienced earlier in life. They indicated the intensity by putting a mark on the Borg CR 10 scale, numbered 0 to 10 [18].

#### Flow experience

In order to assess a participant`s flow experience we used a Swedish translation of Jackson`s validated short (nine items) State Flow scale [19]. Participants completed the questionnaire immediately after each scenario. The items were answered to what extent they agreed or not by putting a mark on a 10 point VAS-scale ranging from “not at all” to “very much”. From these answers we calculated, as recommended by Jackson, a short version flow score.

#### Number of interventions by the trainer per minute

A research assistant, who was not involved in the scenario, counted the number of interventions/interruptions the trainer made to provide additional clinical information in real time during each scenario. Results were confirmed by two raters counting from the video recordings. The number of interventions was divided by the total time in minutes of each scenario.

#### Trainees’ evaluation of the training

The trainees were asked in an exit questionnaire to respond to the following statements: “List the three best elements of/moments in the training”, and “list three elements of/moments in the training that need to be improved”.

#### Power analysis

The power analysis was an assumption regarding mental strain. A sample size of 17 teams in each group was calculated to have 80% power to detect a difference in means of 2.10 assuming that the common standard deviation is 2.10 using a two group t-test with a 0.05 two-sided significance level.

#### Statistical analyses

In comparison between the two fidelities LFM and HFS regarding clinical performance independent t-test was used and when controlling for cases (asthma and sepsis) a two-way factorial analysis of variance (ANOVA) was performed. The statistical unit for these analyses was the team.

The individual trainee’s assessment of mental strain and experience of flow were analysed using a two-way factorial ANOVA with the factors fidelities and roles.

Forward stepwise regression analysis was performed to evaluate the extent to which variations in mental strain and flow experience could be explained by age, sex, profession, earlier training with a simulator and role. Estimates from the statistical tests as mean values, regression coefficient (b), standard errors (SE) and 95% confidence intervals are presented in the tables along with p-values. P < 0.05 was considered statistically significant.

To compare fidelities for the five trainers with respect to mental strain, experience of flow and number of interventions per minutes a mixed linear model involving fidelities as the within-subject variable was used.

### Software used

Statistica 10.0, StatSoft® , Inc. Tulsa OK, USA and SAS® System 9.1, SAS Institute Inc., Cary, NC, USA.

## Results

**Figure 2:**
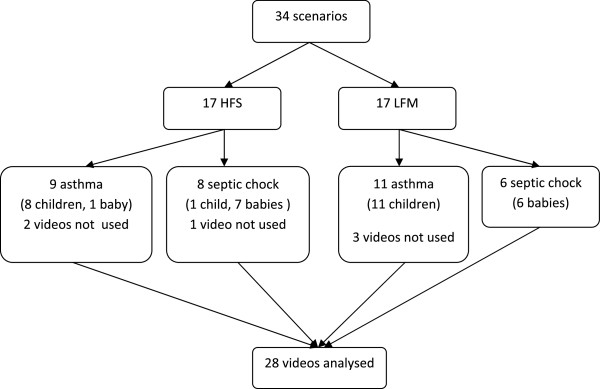
**Lost videos and cases.**

In total, 168 individuals participated, yielding 225 trainees’ occasions during 34 sessions. See Figure 1 for their age, sex and profession as well as for the number of questionnaires and videos received and analysed. In all, there were five trainers. The first 16 sessions were performed using a LFM. Subsequently two sessions were performed with a HFS used in the first, and a LFM used in the second session. Finally 16 sessions with a HFS were performed. For lost videos and cases, see Figure 2.

### Clinical performance

The times from the start of each scenario to the interventions to prescribe and to deliver oxygen are presented in Table 1. The time to prescribe oxygen was numerically longer in the HFS-scenarios, but not statistically significant. Time to deliver oxygen was longer when trainees were using an HFS.

**Table 1:** **Performance and individual experiences in low- and high-fidelity conditions**

Performance/individual experiences	Subjects		Low-fidelity (LFM)			High-fidelity (HFS)			
			N	Mean (SE)	95% CI	n	Mean (SE)	95% CI	p-value
**Oxygen prescribed (s)**	Team		14	57.4 (7.9)	41.1 – 73.8	14	79.7 (7.9)	63.4 – 96.1	n.s. (0.058)
**Oxygen delivered (s)**	Team		14	66.9 (10.4)	45.5 – 88.2	14	105.4 (10.4)	84.1 – 126.8	0.014
		All	86	3.6 (0.3)	3.1 – 4.1	73	4.0 (0.3)	3.5 – 4.5	n.s.
**Mental strain (0–10)**		Leader	15	4.0* (0.5)	3.1 – 4.9	14	4.6* (0.5)	3.7 – 5.6	n.s.
	Trainee	Follower	71	3.3* (0.2)	2.8 – 3.7	59	3.4* (0.2)	2.9 – 3.8	n.s.
**Flow experience (0–100)**		All	88	59.1 (1.9)	55.3 – 62.9	75	54.3 (2.1)	50.2 – 58.3	n.s.
		Leader	16	58.9 (3.4)	52.0 – 65.6	14	51.7 (3.7)	44.4 – 59.0	n.s.
	Trainee	Follower	72	59.4 (1.6)	56.2 – 62.7	61	56.9 (1.8)	53.4 – 60.4	n.s.
			**N**	**Estimated mean (SE)**	**95% CI**	**n**	**Estimated mean (SE)**	**95% CI**	**p-value**
**Frequency of interventions** **(min** ^**−1**^ **)**	Trainer		17^†^	2.4 (0.1)	2.0 – 2.8	5^†^	1.3 (0.1)	0.9 – 1.7	<0.001
**Mental strain (0–10)**	Trainer		17^†^	5.2 (1,1)	2.2 – 8.2	5^†^	2.7 (1.1)	^‡^	<0.001
**Flow experience (0–100)**	Trainer		17^†^	58.5 (5.0)	43.2 – 73.9	5^†^	66.9 (5.0)	51.6 – 82.2	0.004

*Analyzing LFM and HFS comparing leaders and followers, there was no significant difference using LFM, but a significant difference (p = 0.019) between leaders and followers, where leaders experienced higher mental strain using HFS. The numbers of questionnaires vary due to; for the team: lost videos and for trainees: missing values. ^†^The number of trainers was five, but the number of sessions 17. ^‡^Due to the small degrees of freedom the estimated 95% CI is not relevant for this estimate.

The p-value for oxygen prescribed was p = 0.072 and for oxygen delivered p = 0.017 after adjusting for case in the scenario (asthma or sepsis); thus the results were not influenced to a great degree by case.

### Mental strain and experience of flow

The results shown for trainees are from each individual’s first training session. Trainees’ individual experiences did not differ between the two fidelities, Table 1. Separating the two roles, leaders reported higher mental strain using the HFS than followers did, Table 1, note.

Analysing mental strain and flow experience for LFM and HFS together, we found that leaders reported higher mental strain than followers did, but flow experience did not differ between the two roles, Table 2.

**Table 2:** **Results for leaders’ and followers’ mental strain and flow experience for both low- and high-fidelity conditions**

Individual experiences	Leaders			Followers			
	n	Mean (SE)	95% CI	n	Mean (SE)	95% CI	P
**Mental strain (0–10)**	29	4.3 (0.3)	3.7 – 5.0	130	3.3 (0.2)	3.0 – 3.6	0.007
**Flow experience (0–100)**	30	55.2 (2.5)	50.2 – 60.2	133	58.2 (1.2)	55.8 – 60.5	n.s.

The trainers’ reported lower mental strain and their experience of flow was higher when trainees used an HFS, Table 1.

### Number of trainer interventions per minute

Data for the number of interventions per minute is shown in Table 1.

### Regression analysis

To analyse other possible explanations for our findings, we performed a stepwise regression for the trainees’ first session using the dependent variables mental strain and flow experience respectively and using the independent variables sex, age, professions, earlier training with a simulator and leader-follower, Table 3. Profession accounted for about 7% of the variation (R^2**)**^ in mental strain. Age and professions together explained about 10% of the variation (R^2)^ in flow.

**Table 3:** **Regression analysis, dependent variable flow and mental strain**

	Flow				Mental strain			
Independent variables	Estimate	SE	p	95% CI	Estimate	SE	p	95% CI
**Constant**	39.2				4.08			
**Age**	0.39	0.11	0.001	0.17 – 0.60				
**Nurses versus physicians**	5.72	2.31	0.014	1.16 – 10.27	−0.77	0.30	0.011	−1.36 – -0.17
**Nurse assistants versus physicians**	8.37	3.36	0.014	1.74 – 15.0	−1.52	0.44	0.001	−2.39 – -0.64

### Trainees’ evaluation of the training

After using an LFM none of the trainees expressed that the simulator was the best element of the training and 12% (11 of 95 comments) thought that realism was the best element. In contrast, after using an HFS, 20% (23/113) of the trainees expressed that the simulator was the best element and 35% (40/113) thought that realism was the best element.

For LFMs, 19% (13/68) of the trainees mentioned that the simulator needed improvement and 3% (2/68) that the training’s realism did. For HFSs, 10% (7/72) of the trainees stated that the simulator was in need of improvement and just 1% (1/72) indicated that realism did.

### Missing values

There were 2.1% and 2.4% overall data missing from the flow and mental strain questionnaires, respectively.

## Discussion

In this study we evaluated whether high or low levels of equipment fidelity (HFS, LFM) made a difference in trainees’ and trainers’ performances and in individual experiences during in situ simulation based team training for a paediatric emergency.

The time that elapsed before trainees delivered oxygen was longer using an HFS than it was when they used an LFM. Team leaders using a HFS, reported a higher level of mental strain than team followers did. The trainees’ evaluations of the training showed a more positive attitude towards training with an HFS. The frequency of trainers’ interventions was lower together with a lower mental strain and higher flow experience using a HFS.

To our knowledge this study is unique in its approach, which considers both trainees’ and trainers’ performance and individual experiences during the early phase of teamwork training using high- or low fidelity equipment keeping other factors constant.

### Trainees’ performance

In this study we found differences in trainees’ performance between the two levels of fidelity. During training with the HFS, trainees’ actions were time consuming and resembled the authentic clinical context in contrast to the training with an LFM, during which the trainer orally conveyed physiologic data, precluding trainees from reading and interpreting figures on monitors. The longer time to perform the measured interventions using an HFS might be interpreted as a consequence of the higher degree of realism indicated in trainees’ comments.

### Trainees’ individual experiences

The level of trainees’ mental strain and their flow experience did not differ between LFM and HFS training sessions. Since there were no extreme values reported a ceiling effect seems unlikely. The absent difference might be a result of the professional staffs’ ability to engage and involve in the scenario, regardless of equipment fidelity. It might also be a result of the fact that when working with a child, only a few people can stand very close to the patient, and working in the periphery supplying the closer ones with equipment might also reduce the impact of the fidelity level.

Scrutinizing individual experiences using an HFS, leaders scored a higher mental strain than followers did, confirming findings in an earlier study [15]. In a study on simulated neonatal resuscitation Finan et al. found no differences between low- and high-fidelity neonatal simulators concerning trainees’ performance, non-technical skills, subjective short-term stress or salivary cortisol [20]. This lack of difference in trainees’ stress response is coherent with the lack of difference in trainees’ short-term mental strain in our study. Finan et al. found that the leader role correlated with increased salivary cortisol levels. This also aligns with our finding of higher mental strain among leaders. When using an LFM leaders did not score significantly higher on short term mental strain than followers did, which might be a token of lower degree of sense of realism.

To shed light on other possible explanations for our findings, we performed a regression analysis for both mental strain and flow experience. None of the variation in results was influenced by the sex of the trainees, again concurring with earlier findings concerning team leaders and followers during team training [15].

### Trainers’ performance

As expected, the frequency of trainers’ interventions when teams used an HFS was much lower than when they used an LFM. In LFM sessions, the trainer had to deliver physiologic values to the team continuously, and it is reasonable to suggest that this condition increased the load on the trainer’s working memory. Using a HFS could release capacity for a closer observation of individual behaviours and for a more accurate preparation of feedback for the trainees and may thus involve the trainers more efficiently [17].

### Trainers’ individual experiences

In general, training is a complex task. During a scenario the trainer must pay full attention to the process and progress of the scenario, the behaviour of the trainees and simultaneously prepare the feedback that the trainees receive immediately after the scenario. That an HFS supplying physiologic data may reduce the load on an instructor’s working memory is a reasonable explanation for the instructors’ lower mental strain when using HFS.

The experience of flow for the trainer was higher with the HFS. This may be a token of the balance between the increasing challenge a trainer encounters and his or her increasing competence [21]. Flow is a strong motivating force and research suggests that there is a relationship between flow and skills development. The higher level of flow may be valuable for the trainer’s progress in his or her role as such [16]. Harder et al. discussed instructors’ perceptions of what it is like to engage in high-fidelity simulation, they concluded that how the instructors feel about their ability to facilitate has also a perceived effect on students’ learning [22].

### Limitations

The results of this case control study might be affected by the long duration of data sampling (six semesters) and by the risk of subsequent changes in knowledge, skills and attitudes between the first and the last session. The trainers were not the same in all sessions; however they were always physicians familiar with the training concept, so any personal influences the trainer might have exercised on the training were limited.

As seen in Figure 1, seven trainees from six scenarios rejected to use the videos. Of the six scenarios not included, three used LFM and three HFS, thus equally distributed. The performance in these videos might have been substandard and their exclusion influence results.

Due to logistics at the ward some participants trained more than once, and some had taken part in other kinds of training. As we not were interested in how these experiences might change over time and scenarios, we compared the participant´s first experience of each type of fidelity. We also included “earlier training with a simulator” in the regression analyses. The results showed no contributions of earlier training to the variation in mental strain or to the variation in flow.

If training improves clinical performance, the time to prescribe and deliver oxygen would decrease after training. Although the training sessions (with one exception) using the HFS were carried out after the LFM sessions, the times to prescribe and deliver oxygen were longer in the HFS scenarios. This finding disaffirms a major effect on team performance of earlier training.

## Conclusions

Our findings strengthen conclusions from previous research that equipment fidelity is not the only factor that matters; a well-designed curriculum works well with low equipment fidelity. Although, our data suggest that high fidelity equipment might be of benefit for the learning process. Future research may clarify whether high fidelity equipment shortens the learning curve.
